# Methylphenidate treatment of attention deficit hyperactivity disorder in young people with learning disability and difficult-to-treat epilepsy: Evidence of clinical benefit

**DOI:** 10.1111/epi.12399

**Published:** 2013-10-15

**Authors:** Tangunu Fosi, Maria T Lax-Pericall, Rod C Scott, Brian G Neville, Sarah E Aylett

**Affiliations:** * The National Centre for Young People with Epilepsy (NCYPE)Surrey, United Kingdom; † Great Ormond Street Hospital for Children NHS TrustLondon, United Kingdom; ‡ Neurosciences Unit, UCL Institute of Child HealthLondon, United Kingdom; § South London and Maudsley NHS Mental Health TrustLondon, United Kingdom

**Keywords:** Methylphenidate, Attention deficit hyperactivity disorder, Refractory epilepsy, Learning disability, Children

## Abstract

**Purpose:**

To establish the efficacy and safety of methylphenidate (MPH) treatment for attention deficit hyperactivity disorder (ADHD) in a group of children and young people with learning disability and severe epilepsy.

**Methods:**

This retrospective study systematically reviewed the case notes of all patients treated with methylphenidate (MPH) for Diagnostic and Statistical Manual of Mental Disorders, Fourth Edition (DSM-IV) ADHD at a specialist epilepsy center between 1998 and 2005. Treatment efficacy was ascertained using clinical global impressions (CGI) scores, and safety was indexed by instances of >25% increase in monthly seizure count within 3 months of starting MPH.

**Key Findings:**

Eighteen (18) patients were identified with refractory epilepsies (14 generalized, 4 focal), IQ <70, and ADHD. Male patients predominated (13:5) and ADHD was diagnosed at a median age of 11.5 years (range 6–18 years). With use of a combination of a behavioral management program and MPH 0.3–1 mg/kg/day, ADHD symptoms improved in 61% of patients (11/18; type A intraclass correlation coefficient of CGI 0.85, 95% confidence interval [CI] 0.69–0.94). Daily MPH dose, epilepsy variables, and psychiatric comorbidity did not relate to treatment response across the sample. MPH adverse effects led to treatment cessation in three patients (dysphoria in two, anxiety in one). There was no statistical evidence for a deterioration of seizure control in this group with the use of MPH.

**Significance:**

Methylphenidate with behavioral management was associated with benefit in the management of ADHD in more than half of a group of children with severe epilepsy and additional cognitive impairments. Eighteen percent had significant side effects but no attributable increase in seizures. Methylphenidate is useful in this group and is likely to be under employed.

Children with epilepsy show a diminished capacity for sustained attention (Besag, 2002; Sanchez-Carpentero & Neville, 2003; Torres et al., 2008). Permanent factors, such as structural brain abnormality or genetic factors, may underlie the reduced attention in some cases (Kolk et al., 2001; Sanchez-Carpentero & Neville, 2003; McLellan et al., 2005; Hermann et al., 2007; Torres et al., 2008; Bechtel et al., 2009). Yet, there is also a large number of patients in whom easily modifiable factors that impair attention can be identified. Common factors include: the use of particular antiepileptic drugs (AEDs), frequent periictal periods, and dysphoric emotional states (Weinberg & Emslie, 1991; Neville, 1999; Besag, 2002; Sanchez-Carpentero & Neville, 2003; Torres et al., 2008; Hamoda et al., 2009). Optimal management therefore addresses seizure-related, AED-related, and psychological factors that impair attention. It is advisable to use an AED that combines effectiveness with a low risk for inducing inattention, and to avoid polypharmacy (National Institute for Health & Clinical Excellence, 2008, 2012). Where attention difficulties persist despite the above strategies, behavioral and pharmacologic treatment for attention deficit hyperactivity disorder (ADHD) should be considered.

ADHD is an important cause of inattention and reduced quality of life in children with epilepsy, affecting 12–20% (Steffenburg et al., 1996; Davies et al., 2003; see Kaufmann et al., 2009 for review). Among children with epilepsy, ADHD is more common and more severe in the subset who have complex epilepsy, defined as epilepsy associated with additional neurologic impairments (Hoare, 1993; Sherman et al., 2007). Making the diagnosis of ADHD in children with epilepsy is clinically important, as it is now recognized that specifically treating ADHD improves attention in children with epilepsy (see Torres et al., 2008 for review). The treatment of ADHD comprises behavioral intervention and, if needed, a stimulant such as methylphenidate (MPH) or dexamphetamine; or a noradrenaline re-uptake inhibitor such as atomoxetine (MTA Co-operative group, 1999; Barkley, 2003; National Institute for Health & Clinical Excellence, 2008, 2012; Baptista-Neto et al., 2008; Gonzalez-Heydrich et al., 2010). The most commonly used stimulant, MPH, appears to improve ADHD symptoms in 70–80% of children with well-controlled epilepsy and ADHD (Gross-Tsur et al., 1997; for review see: Tan & Appleton, 2005; Koneski et al., 2011)—a similar efficacy to that found in the general population. There are case series in which seizure exacerbation has been reported with use of MPH (Gross-Tsur et al., 1997; Hemmer et al., 2001; Gonzalez-Heydrich et al., 2004). There are, however, no randomized controlled trials that show an increased seizure frequency or severity in patients with epilepsy who are treated for ADHD with MPH. Current evidence from retrospective chart studies, open-label trials, and controlled trials does not support the view that MPH increases seizure frequency in well-controlled epilepsy (Gross-Tsur et al., 1997; Baptista-Neto et al., 2008; Torres et al., 2008).

There is, however, less evidence regarding the use of MPH in children with difficult-to-control epilepsy and learning disability (LD) (Simonoff et al., 2006) (LD refers to permanent impairments of cognitive function having onset in childhood, a term equivalent to ‘mental retardation’ Diagnostic and Statistical Manual of Mental Disorders, Fourth Edition [DSM IV] or intellectual ‘disability’ [DSM V] in the North American usage). Population prevalence studies of ADHD in this patient group are unavailable, but in tertiary clinic populations of children with epilepsy that is difficult-to-control and associated with LD, ADHD may be present in 30–40% (Hempel & Frost, 1995; Semrud-Clikeman & Wical, 1999; Dunn et al., 2003). There is clinical uncertainty regarding the efficacy of the pharmacologic management of this patient group (Torres et al., 2008). There are no published randomized controlled trials (RCTs) or cohort studies specifically addressing the use of MPH in this group. These patients are commonly excluded from major MPH treatment studies (MTA Co-Operative group, 1999; Abikoff et al., 2004). There are concerns about MPH aggravating seizures, and whether adverse effects are increased in children with complex epilepsy (Gross-Tsur et al., 1997; Gonzalez-Heydrich et al., 2010). This group of children with difficult-to-control epilepsy has additional impairments including psychiatric syndromes, neurologic deficits, and LD.

This study helps to bridge this gap. The clinical experience with MPH for treating ADHD in children with severe LD and difficult-to-control epilepsy over a 7-year period (1998–2005) at a national center for epilepsy and LD was reviewed. MPH treatment efficacy was assessed, and the incidences of deterioration of seizure frequency and medication discontinuation due to adverse effects were monitored.

## Methods

A retrospective case note review was conducted for the period 1998–2005 at a residential center providing specialist multidisciplinary (including educational, psychological, and medical) care for children and young people with complex epilepsy and LD (www.youngepilepsy.org.uk). The medication database at Young Epilepsy (formerly known as the National Centre for Young People with Epilepsy, NCYPE) covers all medications given to patients: MPH was used for ADHD (n = 18). Clinical notes and medication administration records ascertained MPH dose data, the timing of medication discontinuation, and the reason for this (therapeutic nonresponse and adverse reactions).

### Clinical assessment and diagnosis of ADHD and learning disability

The clinical case notes for the 18 patients were reviewed by the first author, and the following details were extracted: brain neuroimaging result, category of LD, age at diagnosis of epilepsy, type of epilepsy, seizure frequency prior to and on MPH treatment, chronological age at ADHD diagnosis, additional psychiatric comorbidity, treatment dose of MPH administered for ADHD, treatment response to MPH, duration of MPH treatment, and the occurrence of adverse reactions leading to the discontinuation of MPH. Instances where MPH was discontinued owing to intolerable adverse effects can be ascertained reliably, and thus were chosen to index the tolerability of MPH in this sample. Minor MPH adverse effects that did not result in medication discontinuation were expected to be a less reliable indicator of the tolerability of MPH in this sample, and were therefore not examined.

The diagnosis of ADHD was made following referral to a child and adolescent psychiatrist, and a team comprising a clinical psychologist, a pediatric neurologist, an epilepsy nurse specialist, an educator, and the child’s carer all provided reports on the child as part of routine clinical management. The assessments of this multidisciplinary team formed the basis for making a clinical diagnosis of ADHD according to DSM-IV-TR criteria (American Psychiatric Association, 2000). The diagnosis requires six or more inattentive symptoms; or six or more hyperactive-impulsive symptoms, with ADHD being classified as inattention-predominant, hyperactivity-predominant, or combined type. Behavioral observations on each child were combined by intersection (‘AND’), that is, parent/care and teacher reports had to concur for a given DSM-IV criterion (Rowland et al., 2008). The multidisciplinary team’s contemporaneous clinical note records of the child’s response to treatment were examined systematically.

The procedure used for subtype assignment in different studies has been shown to have a significant influence on the relative prevalence of ADHD subtypes reported (Rowland et al., 2008). Specifically, single-informant teacher reports increase the likelihood of diagnosing predominantly inattentive ADHD; single-informant parent reports increase the likelihood of diagnosing predominantly hyperactive-impulsive ADHD; and combined parent and teacher reports increase the likelihood of diagnosing combined ADHD. Combined informants’ observations may be combined by union (‘OR’) or intersection (‘AND’) rules, with the choice of method influencing the ADHD subtype diagnosed. Union rules assess the criteria for an ADHD subtype in *either* the parent or teacher report; whereas intersection rules require *both* parent and teacher reports to concur on a subtype criterion for it to be valid in the particular child. Union rules result in an increased diagnosis of combined ADHD, whereas intersection rules increase the diagnosis of inattentive ADHD (Rowland et al., 2008).

Behavioral management as part of the general approach to this group of children and young people was the first line of management, and if this was unsuccessful in improving the patient’s functional level in relation to their ADHD, stimulant treatment was added. Behavioral interventions alone were not sufficient to control the ADHD symptoms of any of the patients in this study. Consequently, medication was instituted as add-on to behavioral intervention in all patients in this study. Prior to start of the medication, the parents of the child were contacted and informed consent obtained after face-to-face discussion. MPH was the stimulant used in 17 children after behavioral management. In another child (patient 17), dexamphetamine was used after behavioral management, but owing to a lack of success this was changed to MPH. Data were therefore available for 18 patients on MPH. MPH was titrated to within a range of 0.5–1 mg/kg/day against the patient’s ADHD symptoms and function as observed by carers and educators, until a stable improvement was obtained.

### Psychological assessment and behavioral interventions

At NCYPE each child was seen on admission and at regular follow-up by a clinical psychologist who assessed their learning ability on clinical grounds and with psychometric tests when the child was able to cooperate. The psychologist’s choice of test instrument was made on clinical grounds, and included the Wechsler Intelligence Scale for Children-revised (WISC-R) (Wechsler, 1974) or Stanford–Binet test (Thorndike et al., 1986). The clinical classification of learning disability at the center was: mild (IQ 50–70), moderate (IQ 35–49), or severe (IQ 20–34) (American Psychiatric Association, 2000, 2013). The clinical psychologist’s case note records and the cognitive test results were reviewed.

Behavioral management was the first-line intervention for ADHD, teaching the child response delay techniques. A comprehensive, individually adapted behavioral intervention plan was implemented for each child. The following interventions were affected: increased environmental structure; one-step task instructions with repeat and visual reinforcement; immediate feedback for appropriate and problem behaviors; and operant conditioning, involving coupling rewards for appropriate behavior (e.g., impulse control) with loss of rewards for problem behaviors. Each child received 24-h supervision from adult carers and education staff.

### Clinical assessment and diagnosis of epilepsy

Where possible the patients’ epilepsy syndromes were classified according to the International League Against Epilepsy framework (Engel, 2006). Active epilepsy was defined by the occurrence of at least one seizure in the 2-year period preceding the initiation of pharmacologic treatment for ADHD. Refractory epilepsy was active epilepsy in which seizures occurred at least weekly despite the adequate trial of at least two appropriate AEDs, or active epilepsy despite cotherapy on two or more AEDs (Arroyo et al., 2002; Mohanraj & Brodie, 2006; Kwan et al., 2010). The total number of AEDs that each patient received concurrently with MPH and prior to MPH was recorded.

Seizure frequency data were obtained from the routine daily seizure frequency chart. The baseline monthly seizure frequency at the initiation of first-line stimulant treatment was calculated as the mean monthly seizure frequency for the preceding 6 months (epoch A). The mean monthly seizure frequency for the 3 months after the initiation of first-line stimulant treatment was calculated (epoch B). For patients who received less than 3 months of stimulant treatment, epoch B was calculated as the mean monthly seizure frequency for the period of treatment. Where MPH was taken for <3 months, epoch B was the average over that period. A 25% or greater rise in mean monthly seizure count between the two epochs (A and B) was regarded as significant.

We could find no published value of ideal cutoff that defines a significant increase from baseline seizure frequency and that is uniformly applicable across the population of children with refractory epilepsy. The cutoff of a 25% or greater rise in mean monthly seizure count was chosen to assess seizure deterioration on MPH in this study for two reasons. First, the 25% cutoff provides quantification of the relative change in seizure control in a manner commonly understood in clinical epilepsy research (Hosain et al., 2006). Second, because the patients in this sample have a wide range of seizure frequencies, this calculation provides for each patient’s on-treatment period to be expressed relative to their pretreatment period, with the latter serving as a control. This simple index provides a summary view of the relative change in seizure frequency over a 3-month period from initiating MPH. Time-series modeling methods have been applied for seizure frequency analysis in refractory epilepsy to permit more sensitive analyses (Balish et al., 1991; Pujar et al., 2010). Given the potential for recording bias that exists with retrospective data, these methods would, however, perform suboptimally in this context, in contrast to the relatively simple method used in this study.

### Clinical global impressions scoring

The response of ADHD symptoms to MPH treatment was scored by four clinicians who independently reviewed each child’s records while blinded. The blinding procedure used was the following: one author who did not participate in the scoring (TF) photocopied the case notes, removing all personal identifiers (name, date of birth, hospital number) from the resulting record. This record was provided to each of the remaining four authors to score. The four assessors were three consultant pediatric neurologists (SA, BN, and RS) and a consultant child psychiatrist (TLP).

The score used was a modified version of the global improvement item of the Clinical Global Impressions (CGI) scale (National Institute of Mental Health, 1970). The CGI scale has been used reliably in psychiatry research on children to assess clinical response to treatment (Santosh et al., 2006). The global improvement item of the CGI, is a seven (7)-point Likert scale (1 = very much improved to 7 = very much worse). The CGI was scored by each of the four assessors, with a dichotomization of each assessor’s result into an outcome: “improved” (scores 1–3) or “not improved” (scores 4–7). The assessment data for each subject by the four independent assessors were combined to classify the therapeutic outcome for the patient. A patient was assigned to the treatment response group (“improved” or “not improved”) to which the majority of assessors had assigned him/her. The treatment response was classified as “equivocal” if the assessors were equally likely to assign the patient to either outcome group. There were, consequently, three groups of primary treatment response to MPH: *responders*, *nonresponders* and *equivocal*. The response rate was the number in the *responder* group as a proportion of the total number of subjects. The formal expression used was:

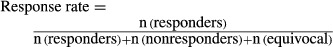


The mean agreement between the ratings made by the assessors was also quantified. This was done using the type A intraclass correlation coefficient (ICC) for absolute agreement between the assessors’ CGI ratings. This technique is an application of a two-way mixed model (Landis & Koch, 1977; Shrout & Fleiss, 1979).

## Results

### Patient characteristics

#### Group characteristics (ADHD)

**Figure 1:**
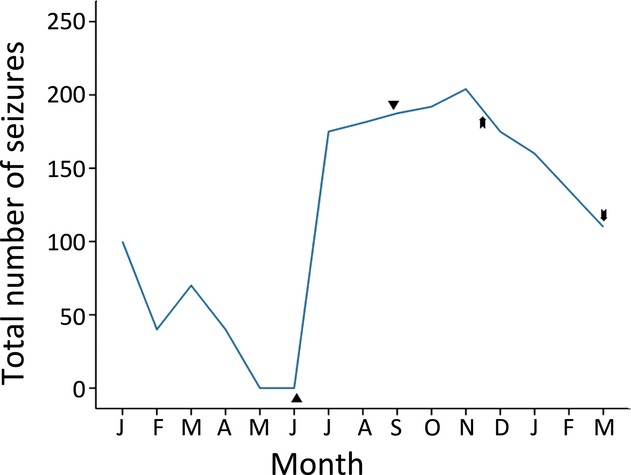
Seizure frequency of patient 7 on MPH and dexamphetamine. ▲MPH started (0.1 mg/kg/day). ▼MPH stopped (0.1 mg/kg/day). 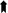 Dexamphetamine started (0.2 mg/kg/day). 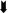 Dexamphetamine stopped (0.4 mg/kg/day).

Eighteen patients with epilepsy (13 boys, 5 girls: all with combined-type ADHD) were treated with MPH, of a population of 198 children admitted to the center between 1998 and 2005. The median age at ADHD diagnosis was 11.5 years (range 6–18 years) and the median duration of stay at the center prior to the diagnosis of ADHD being made was 1 year (range 0.1–11.0 years; mean 1.6, standard deviation [SD] 2.9 years: Fig. 1). The median duration of treatment with MPH was 12 months (range 2–108 months), and the median maximum dose of MPH attained during treatment was 0.5 mg/kg/day (range 0.2–1.2 mg/kg/day). Nearly all patients (16/18) had at least one other DSM-IV psychiatric diagnosis. The psychiatric comorbidities seen, in descending frequency were the following: oppositional defiant disorder, autism spectrum disorder, conduct disorder, mood disorders, and tic disorder. All patients had LD. This was severe in two thirds (12/18), with the remaining patients being equally distributed between moderate LD (3/18) and mild LD (3/18) groups. These are summarized in Tables 1 and 2.

**Table 1:** Individual patient characteristics (ADHD)

Patient	Sex	Learning disability severity	Age at ADHD diagnosis (years)	Psychiatric comorbidity	Responder to MPH	Max MPH dose (mg/kg/day)	Duration on MPH (month)	Cessation for adverse drug event
1	M	Severe	14	2, 3	Yes	0.3	30	No
2	F	Mild	11	2	Yes	0.6	32	No
3	M	Severe	17		Yes	0.5	24	No
4	M	Severe	8	1, 2	Yes	0.6	2	No
5	F	Severe	12	1, 2, 3	Yes	1.2	4	No
6	F	Severe	11	2	No	0.2	3	Yes
7	M	Severe	6	2	Yes	0.5	3	No
8	M	Mild	10		Yes	0.5	6	No
9	M	Mild	12	2, 3	No	0.3	2	Yes
10	M	Severe	11	1	No	0.5	12	No
11	M	Severe	9	2, 4	Yes	0.4	108	No
12	M	Severe	16	3	No	0.8	21	No
13	M	Severe	13	1, 2	No	0.8	4	Yes
14	M	Moderate	12	1	Yes	0.6	84	No
15	M	Moderate	17	2	Yes	0.5	12	No
16	F	Severe	18	2	Equivo.	0.8	15	No
17	M	Severe	9	2	Yes	0.5	69	No
18	F	Moderate	11	2	Equivo.	0.3	2	No

Psychiatric comorbidity: 1, autism spectrum disorder; 2, oppositional defiant disorder; 3, mood disorder; 4, tics.

**Table 2:** Group characteristics (ADHD)

	Median	Mean	SD	Range
Age at ADHD diagnosis (years)^a^	11.5	12.1	3.3	6–18
Duration of MPH treatment (months)	2	24	31	2–108
Maximum MPH dose (mg/kg/day)	0.5	0.6	0.2	0.2–1.2
Duration before ADHD diagnosis (years)	1	1.6	2.9	0.1–11
				Number of subjects
Sex				
Male				13
Female				5
ADHD type				
Combined				18
Inattentive-predominant				0
Hyperactive-predominant				
Comorbidity				
Oppositional defiant disorder (ODD)				13
Autism spectrum disorder				4
Emotional disorder				
Tics				1
Learning disability				
Mild (IQ 50 – 70)				3
Moderate (IQ 35 – 49)				3
Severe (IQ 20 – 34)				12

a Same as age at start of MPH.

#### Epilepsy

The median age at onset of epilepsy was 2.3 years (range 0.5–11 years), with epilepsy having started at or before age 6 years in 16 cases and in the two remaining cases, at 9 and 11 years of age.

Fourteen patients had generalized epilepsy, meeting the clinical criteria for epilepsy within the Lennox-Gastaut spectrum (13) and Doose syndrome (1). Four patients had symptomatic focal epilepsy: two had bilateral hippocampal sclerosis with temporal lobe epilepsy; one had tuberous sclerosis, and the other had a congenital noncommunicating hydrocephalus.

All patients were taking AED treatment for at least 6 months at the time of starting MPH. Across the entire sample, a median of two (2) concurrent AEDs was being received at the initiation of MPH. The median cumulative number of previous AEDs received was 4 (range 2–13 AEDs). All patients with active epilepsy at the initiation of MPH (10 patients, 55%) fulfilled the criteria for refractory epilepsy. For those with treated, inactive epilepsy at the initiation of MPH (eight patients, 45%), seizure-freedom was being maintained on one AED (four patients: 3, 11, 15, and 18) or on two concurrent AEDs (four patients: 1, 5, 9, and 13). Three of the four patients who were maintained seizure-free on monotherapy had met the criteria for refractory epilepsy at some point in the course of their epilepsy. Therefore, all but one patient in the entire sample (patient 18) satisfied the label of refractory epilepsy either preceding or while on ADHD treatment. In summary, the epilepsies in this group of patients were, over their long-term course, clinically difficult-to-treat. We retained all, envisaging the possibility of gaining useful data on the recrudescence of seizures in patients who had gained seizure freedom prior to MPH being initiated.

Tables 3 and 4 summarize the epilepsy-related features of this group.

**Table 3:** Patient characteristics (epilepsy)

Patient	Age at epilepsy onset (years)	Electroclinical epilepsy diagnosis	Neuroimaging	No. of AEDs tried	AED Rx at start of MPH	Epilepsy activity	Avg monthly sz count 6 month before MPH	Avg monthly sz count 3 month after start of MPH	↑ Monthly sz count >25%
1	1	Myoclonic Astatic (Doose syndrome)	Normal	2	LTG	Inactive	0	0	No
					ESX				
2	2.5	Symptomatic focal	Bilateral hippocampal sclerosis	10	OXC	Active RE	29	15	No
					STI				
					CLO				
3	2.5	Lennox-Gastaut	N/A	3	CLO	Inactive, (Prev R.E)	0	0	No
4	2	Lennox-Gastaut	Normal	4	TOP	R.E	85	0	No
					CLO				
5	1	Lennox-Gastaut	Atrophy of corpus callosum	2	TOP	Inactive	0	0	No
					VPA				
6	0.5	Lennox-Gastaut	Normal	11	VPA	R.E	300	300	No
					TOP				
					ZON				
7	0.5	Lennox-Gastaut	Normal	13	VPA	R.E	42	157	Yes
					PHT				
					ESX				
8	4	Symptomatic focal	Cortical tubers	5	VPA	R.E	5	5	No
					LTG				
					CLO				
9	2	Lennox-Gastaut	N/A	4	LTG	Inactive, (Prev R.E)	0	0	No
					CLO				
10	6	Lennox-Gastaut	N/A	4	VPA	R.E	7	7	No
					LTG				
11	2.5	Symptomatic Focal	Bilateral Hippocampal sclerosis	4	LTG	Inactive, (Prev R.E)	0	0	No
12	4	Lennox-Gastaut	Normal	7	VPA	R.E	14	12	No
					LTG				
					ESX				
13	5	Lennox-Gastaut	Normal	5	VPA	Inactive, (Prev R.E)	6	2	No
					LTG				
14	9	Lennox-Gastaut	Normal	2	VPA	R.E	1	0	No
15	0.5	Lennox-Gastaut	Normal	4	None	Inactive, (Prev R.E)	0	0	No
16	2	Symptomatic focal	Hydrocephalus	2	VPA	R.E	4	1	No
					LTG				
17	1	Lennox-Gastaut	Normal	6	CBZ	R.E	29	28	No
					LTG				
					MSX				
18	11	Lennox-Gastaut	Perinatal hypoxic-ischemic cerebral damage	2	VPA	Inactive	0	0	No

CBZ, carbamazepine; CLO, clobazam; ESX, ethosuximide; LTG, lamotrigine; MSX, methsuximide; OXC, oxcarbazepine; STI, stiripentol; TOP, topiramate; VPA, sodium valproate; ZON, zonisamide; PHT, phenytoin; N/A, not available; R.E., refractory epilepsy.

**Table 4:** Group characteristics (epilepsy)

	Median	Mean	SD	Range
Age at onset (years)	2.3	3.1	3.0	0.5–11
Duration of epilepsy (years)	8.2	8.9	4.5	0–16.5
Number of AEDs	2	2	0.8	1–3
Number of failed AEDs	4	5	3.3	2–13
Seizure frequency (number/month)				
Epoch A	1.5	30	73	0–300
Epoch B	1.5	30	73	0–300
				Number of subjects
Type of epilepsy				
Generalized				14
Focal				4
Epilepsy control				
Active epilepsy^a^				10
Inactive epilepsy				8
Neuroimaging				
Normal				9
Abnormalities on MRI				6

AED, antiepileptic drug; MRI, magnetic resonance imaging.

a Active epilepsy: defined as having had at least one seizure in the 2 year period preceding the initiation of methylphenidate.

### Response to MPH treatment

#### Interrater agreement of CGI ratings

CGI ratings by the four independent assessors concurred, with a type A intraclass correlation coefficient (ICC) of 0.85 (95% confidence interval [CI] 0.69–0.94), Table S1. This magnitude of agreement is considered high (Landis & Koch, 1977). CGI ratings from at least three assessors were consistent for 16 of 18 patients: 12 patients received the same CGI rating from all four assessors, and 4 patients received the same rating from 3 of the 4 assessors. The remaining two (2) patients each had conflicting ratings from half of the assessors, so their response was classified as equivocal. Both patients were female.

#### Treatment response rate

The three categories of treatment response were the following: improved (11/18), not improved (5/18), and equivocal (2/18). The positive treatment response rate in this sample of patients with severe epilepsy and learning disability was 61% (11/18, 95% CI 47–75%).

#### Characteristics of responders versus nonresponders

Differences between responders and nonresponders were assessed as a function of gender, LD severity, age at start of MPH, duration of MPH treatment, maximum MPH dose attained, age at epilepsy onset, type of epilepsy (generalized or focal), activity of epilepsy (active or inactive), number of concurrent AEDs, and cumulative number of previous AEDs received. Nonparametric tests were used in view of nonnormal variable distributions: the Mann-Whitney *U* test for continuous variables, and the chi-square test for categorical variables. There was evidence that more nonresponders than responders experienced an adverse effect leading to cessation of MPH (p = 0.02, but p > 0.05 after Bonferroni correction for multiple comparisons). Some evidence existed that nonresponders had a mood disorder more frequently, and received MPH for a shorter time than responders, but these were not statistically significant (p = 0.097 and p = 0.084, respectively).

#### Seizure control on MPH

MPH was stopped in one patient (patient 7) as a result of increased seizure frequency after initiating the drug. This patient met the study’s criterion for significant increase in monthly seizure. The patient’s seizure frequency remained elevated after the discontinuation of MPH. A positive behavioral response to MPH had been obtained, and an increase in ADHD symptoms became increasingly evident off MPH. After a trial of behavioral intervention alone, dexamphetamine was initiated 2 months after stopping MPH. On dexamphetamine the patient’s seizure frequency showed a downward trend during the subsequent 5 months (Fig. 1).

#### MPH discontinuation due to adverse reactions

MPH was discontinued in three patients (patients 6, 9, 13) owing to adverse drug reactions. Patient 6 experienced marked loss of appetite and became highly withdrawn (‘zombie-like’ according to parents and carers). Patient 9 experienced heightened anxiety and deterioration of his oppositional defiant disorder behaviors. Patient 13 became very withdrawn on MPH.

## Discussion

### Clinical response rate and tolerability of MPH

The treatment response rate of 61% (95% CI 47–75%) in this sample of patients with current or previous refractory epilepsy and severe LD is comparable to the rate (54–75%) reported for children with mild-to-moderate learning disability without epilepsy (Handen et al., 1990, 1992). The rate reported in children with well-controlled epilepsy without significant LD is 70% (Gross-Tsur et al., 1997; Tan & Appleton, 2005). Most patients who responded to MPH did so within the relatively narrow dose range of 0.4–0.5 mg/kg/day. The dose range at which MPH produced a stable therapeutic effect on ADHD symptoms ranges in the childhood epilepsy literature from 0.3 to 1 mg/kg/day (Gross-Tsur et al., 1997; Gucuyener et al., 2003). Gross-Tsur et al. (1997) using the lower dose of MPH (0.3 mg/kg/day as a single dose) found that ADHD symptoms improved in 70% of children who had ADHD and epilepsy. The patients in the study of Gross-Tsur et al. mostly had well-controlled epilepsy, were on AED monotherapy, and did not have significant LD. This patient group is different from that reported here. The patient cohort reported here showed additional DSM-IV psychiatric comorbidities in 89% (16/18) (see Table 1). This is similar to the finding by Steffenburg et al. (1996), who found about 90% prevalence of a psychiatric disorder in children with epilepsy and ‘mental retardation’ whose psychiatric conditions could be categorized. We did not find statistical evidence for a relationship between response to MPH and having comorbid oppositional-defiant disorder or comorbid autism spectrum disorder (Santosh et al., 2006). Despite the increased prevalence of additional psychiatric comorbidities in our sample, a clinical response to MPH was obtained in more than half of the patients, and patients who responded to MPH did so within the usual therapeutic dose range.

The study used CGI ratings, which provide information on global improvement in behavior following pharmacologic treatment of ADHD rather than quantifying the response of individual ADHD symptoms. The goal of treatment with MPH was to improve the patient’s function, by enhancing concentration, increasing impulse control, and reducing hyperactivity. The assumption in this study is that the combined effect of MPH on these separate symptoms underlies the improvement in the patient’s overall behavioral profile measured by the CGI. The differential extent to which each separate symptom is altered by MPH can be highly variable in clinical practice (Sonuga-Barke et al., 2008; Takon, 2011). The CGI as implemented in this study would not be informative on this aspect. There is a potential critique that the behavioral improvement measured by the CGI in this study reflects a sedative effect on the treated patients, which renders them unable to manifest problematic behaviors. We do not consider this to have been the case. MPH is not a nonspecific sedative, but there is evidence that MPH lessens the salience within memory of material that would normally be emotionally arousing (Brignell et al., 2007). The resultant lowering of emotional arousal may be misinterpreted as a “sedative effect.” The presumption that the functional improvement in this study emerged from lowering emotional arousal in the patient group would lead logically to a particular prediction. Those patients who developed more marked emotional blunting ought to be likely to demonstrate a concomitant improvement of CGI. During treatment the team specifically looked for the development of either a sedative reaction that impaired the patient’s usual daily activities (significant sedative reaction); or affective reactions (behavioral signs of labile or low mood). These are recognized serious adverse reactions to MPH. The management of these reactions was a dose reduction in the first instance or medication withdrawal if the former did not reverse the effect. This is consistent with the agreed clinical practice for discontinuing medication in the context of intolerable side effects or a lack of efficacy (National Institute for Health & Clinical Excellence, 2008). There were two patients (patients 6 and 13) who developed dysphoric features, and a third patient (patient 9) had marked worsening of anxiety symptoms. These effects are likely related to MPH, since the affective and behavioral changes resolved in all following the discontinuation of the drug. The CGI response for the three patients who showed affective reactions was “nonresponder.” This finding is inconsistent with a hypothesis that lowering the emotional arousal necessarily results in the treatment response falling into the “improved” CGI category.

The rate of MPH treatment cessation due to adverse-effects in this study (3/18: 17%) was similar to the rate (19%) reported by Handen et al. (1991) in their study of children with “mental retardation.” Our experience is that during the treatment of ADHD in patients with difficult-to-control epilepsy, a significant sedative reaction is neither invariable nor typical at standard doses of MPH. The doses of MPH in two of the three patients who developed affective reactions to MPH were relatively low, pointing toward the inference of the affective reactions not being dose-determined. This impression is supported by clinical trial data (Sonuga-Barke et al., 2009). MPH can induce dysphoria in some children, especially those with a genetic susceptibility to low mood (Weinberg & Emslie, 1991). The dysphoric and vegetative symptoms in patients 6 and 13 at low doses of MPH (0.2–0.3 mg/kg/day of MPH) may reflect an existing predisposition. Mood disorders affect 16% of children with epilepsy in population studies (Davies et al., 2003), and up to one third in tertiary clinic populations (Thome-Souza et al., 2004). Establishing the diagnosis of a mood disorder in a child with refractory epilepsy and LD is difficult but important (Thome-Souza et al., 2004; Gonzalez-Heydrich et al., 2007). Both patients were on sodium valproate at the initiation of MPH. This may have exerted a mood-stabilizing effect upon a preexisting depressive disorder, thereby preventing it from coming to clinical recognition. The treatment of ADHD with MPH in this subgroup of patients should be monitored, as a deterioration of attention abilities may ensue on MPH if an anxiety disorder coexists (Goez et al., 2007). The present study evidences a trend for children with a mood disorder to be more frequently nonresponders to MPH.

The view has been advanced that a genetic defect common to ADHD and epilepsy may explain their co-occurrence in patients such as those in the present study (Hamoda et al., 2009). An alternative view is ADHD in the context of refractory epilepsy reflects an alteration of cortical circuits as a result of early brain damage or dysfunction and seizures (Neville, 1999; Sanchez-Carpentero & Neville, 2003; Hermann et al., 2007; Bechtel et al., 2009). The response of ADHD symptoms to MPH in the patient group studied here may suggest that some behavioral manifestations of the long-term effects of seizures on the developing brain can be successfully contained (Neville, 1999).

### MPH treatment and seizure control

The one patient (patient 7, Fig. 1) who experienced an increase in seizures while on MPH did not regain baseline seizure frequency after MPH was withdrawn. It is uncertain if the increased seizure frequency while on MPH in part reflected the patient’s pattern of severe epilepsy, independently of the use of MPH. The study did not find an instance of a patient whose epilepsy was in remission (inactive epilepsy), and which recrudesced following the initiation of MPH. A statistically significant incidence of increase in monthly seizure frequency could not be demonstrated within 3 months of initiating MPH in the 18 patients in this study at a group level (Wilcoxon rank-sum test Z = −0.169, p = 0.866).

It is proposed that larger studies investigate these observations further. The risk of a type 2 error (failing to detect an adverse effect of MPH on seizure control) cannot be discounted in view of the small sample size in the present study.

### ADHD phenomenology in patients with complex epilepsy

A recent study of children with epilepsy, LD, and ADHD also found that the combined ADHD subtype predominates (Gonzalez-Heydrich et al., 2007). In childhood idiopathic epilepsy, ADHD is more often of the inattentive-type, a finding replicated using two different ascertainment methods: single-informant parent reports in Dunn et al. (2003) and combined expert assessment and parent interview in Hermann et al. (2007). In childhood complex epilepsy, the influence of methodology on the classification of ADHD is not known and requires further study.

The present study combined information (expert assessment, parent/carer informant report, and teacher report). This may be expected to increase the diagnosis of combined-type ADHD if union rules are applied. However, intersection rules were used, so the diagnosis of combined type ADHD made in these patients is not expected to simply indicate a methodologic bias. The combined ADHD subtype, which was present in all our patients, is considered the most severe clinically and has the highest rate of psychiatric comorbidity (Faraone et al., 1998).

### Potentially late ADHD diagnosis in this patient group

In this study, children with refractory epilepsy had a mean age at ADHD diagnosis of 12 years (median 11.7 years). This is older than reported in the general population of children diagnosed with ADHD in the United Kingdom (9 years, Parr et al., 2003). This study therefore provides evidence that ADHD is potentially diagnosed late in children with refractory epilepsy compared to their peers in the general population**.**

Factors that may make the diagnosis of ADHD more difficult in refractory epilepsy include the high rate of comorbidities and diagnostic overshadowing (Simonoff, 2003). Comorbidities, for example depression, may produce a similar symptom presentation (Weinberg & Emslie, 1991). Steffenburg et al. (1996) in a Swedish population-based study found that even with parental concern, a large number of psychiatric disorders in children with LD and epilepsy are not diagnosed promptly or remain undiagnosed. There is still a reluctance to diagnose and treat ADHD in these patients (termed “diagnostic overshadowing,” Simonoff, 2003). This may in part reflect the expectation that the complete cessation of seizures must be first achieved, and the concern that seizure frequency may increase on MPH. The first expectation is unlikely to be realized in this population with refractory epilepsy, and the second does not have a strong empirical basis. Diagnostic overshadowing should slowly recede in the light of evidence that indicates the benefit of managing ADHD in children with learning disability and severe epilepsy. It should be emphasized that effective control of ADHD can make a significant contribution to the management, wellbeing, and possibility for relative independence in this highly dependent group.

## Limitations

This was an audit of the clinical use of MPH with structured criteria in place. Although a randomized placebo-controlled study could be performed in a severely affected group with epilepsy the evidence for the positive value in children with milder epilepsy is clear and combined with this study strongly suggests that MPH should not be withheld on theoretical grounds in the severely affected. The theoretical grounds are weak, the response is similar to other studies, and the adverse effects in a minority resolve on withdrawal of MPH.

## Conclusion

There is overwhelming evidence for the benefit of MPH in ADHD in the literature (MTA Co-Operative Group, 1999) and significant evidence that such benefits apply to those with a LD (Handen et al., 1990, 1992) and in general to children with epilepsy (Gonzalez-Heydrich et al., 2010). This small study of children with intractable epilepsy and severe LD fills a gap in showing, perhaps not surprisingly, that similar benefits apply to this group, with a minority incurring adverse effects. The response rate to MPH treatment was 61% in this pediatric sample with ADHD, severe epilepsy, cognitive impairment, and a high rate of psychiatric comorbidity. The rate of adverse effects leading to discontinuation of medication was comparable to that reported in children with primary ADHD. There was no statistical evidence that the treatment of ADHD with MPH directly increased the seizure rate for the majority of patients with refractory epilepsy.

The treatment of ADHD in young people with learning disability and complex epilepsy under specialist care using behavioral interventions and MPH may improve ADHD symptoms without causing a clinically significant increase in seizure frequency. The lack of use of MPH in this group would seem to result from lack of recognition of ADHD and an exaggeration of original concerns that MPH might aggravate seizures. We suggest that, although this is a small study, there is not a strong case for a larger one but that these patients should have the benefit of consideration of this treatment. Children with epilepsy and LD should be evaluated for ADHD, as this may be underrecognized; meanwhile they stand to benefit from multimodal treatment for ADHD. The available data do not exclude a small number of children with epilepsy experiencing an increase in seizures. This study, although not an RCT, would be helpful to clinicians and parents concerned about possible adverse reactions.

## Supporting Information

**Table S1.** Treatment outcome as measured by CGI.
