# Supplementary material for: Methylphenidate treatment of attention deficit hyperactivity disorder in young people with learning disability and difficult-to-treat epilepsy: Evidence of clinical benefit
Source: Epilepsia. 2013 Oct 15;54(12):2071–81. doi: 10.1111/epi.12399 (PMC4209117; doi:10.1111/epi.12399)
Supplement: Supplementary file 1 — Table S1. Treatment outcome as measured by CGI. [file epi0054-2071-sd1.docx]

**Supplemental Table**

**Table 1 :** **CGI Scores and intraclass correlation coefficient**

**CGI Scores**

| **Patient** | **Rater 1** | **Rater 2** | **Rater 3** | **Rater 4** | **AGGREGATED**  **RESPONSE** |
| --- | --- | --- | --- | --- | --- |
| **1** | Y | Y | Y | Y | Y |
| **2** | Y | Y | Y | Y | Y |
| **3** | Y | Y | Y | Y | Y |
| **4** | Y | Y | Y | Y | Y |
| **5** | Y | Y | Y | Y | Y |
| **6** | N | N | N | N | N |
| **7** | Y | Y | Y | Y | Y |
| **8** | Y | Y | Y | N | Y |
| **9** | N | N | N | N | N |
| **10** | N | N | Y | N | N |
| **11** | Y | Y | Y | Y | Y |
| **12** | N | N | N | Y | N |
| **13** | N | N | Y | N | N |
| **14** | Y | Y | Y | Y | Y |
| **15** | Y | Y | Y | Y | Y |
| **16** | N | Y | Y | N | E |
| **17** | Y | Y | Y | Y | Y |
| **18** | Y | N | Y | N | E |

Y= clinical improvement, N = No clinical improvement, E = equivocal

**Legend:**

The intraclass correlation coefficient was calculated using an absolute agreement definition and computed via a two-way mixed effects model . This was a Type A coefficient, where people effects are random and measures effects are fixed. It is assumed that the interaction effect is absent, because it is not estimable otherwise. The **type A intraclass correlation coefficient (ICC) for CGIs was:** 0.848 (95% CI: 0.692 - 0.936).
