# Supplementary material for: Methylphenidate treatment of attention deficit hyperactivity disorder in young people with learning disability and difficult-to-treat epilepsy: Evidence of clinical benefit
Source: Epilepsia. 2013 Oct 15;54(12):2071–81. doi: 10.1111/epi.12399 (PMC4209117; doi:10.1111/epi.12399)
Supplement: Supplementary file 2 [file epi0054-2071-sd2.pptx]

## Slide 1
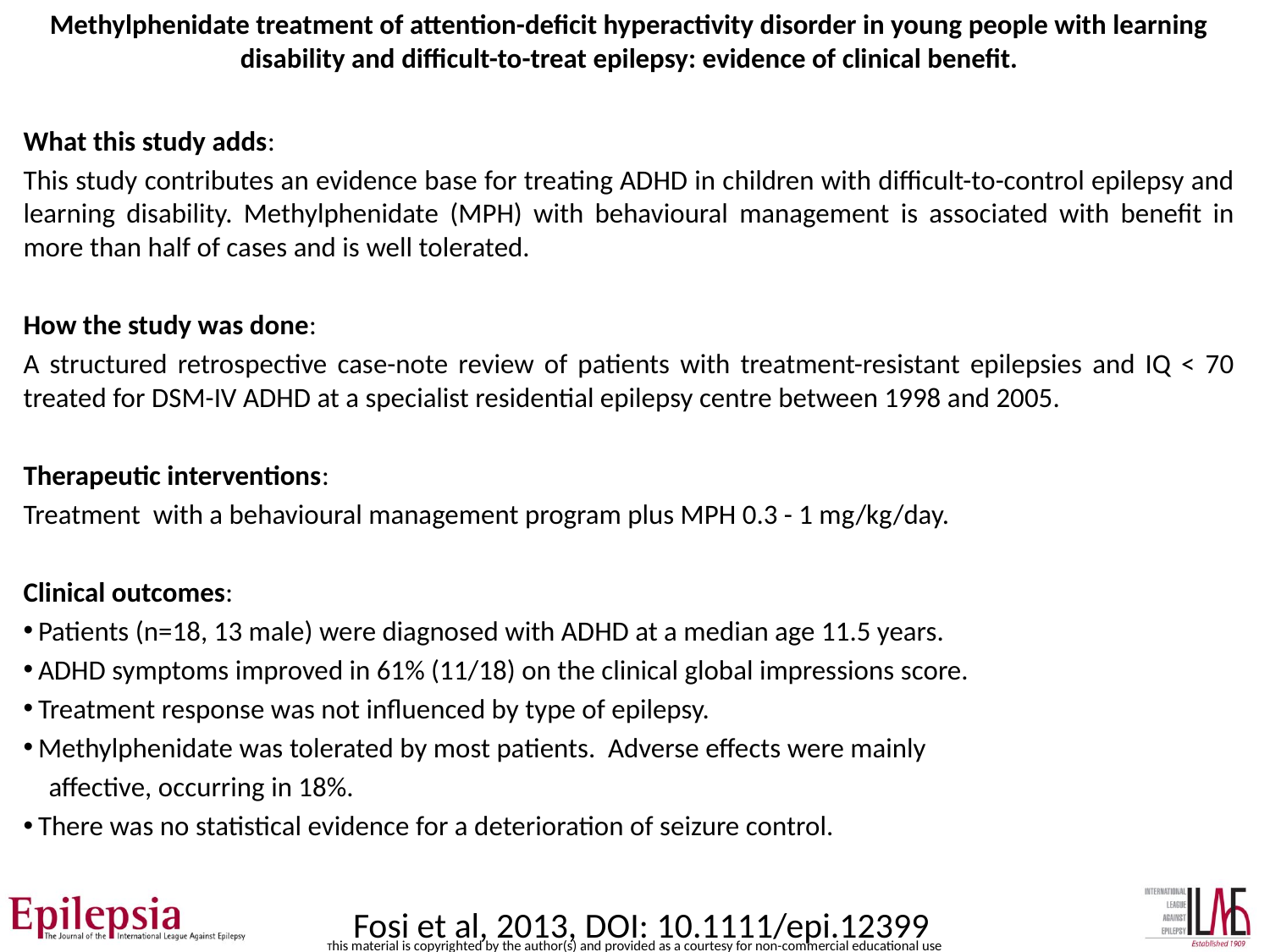

Methylphenidate treatment of attention-deficit hyperactivity disorder in young people with learning disability and difficult-to-treat epilepsy: evidence of clinical benefit.
What this study adds:
This study contributes an evidence base for treating ADHD in children with difficult-to-control epilepsy and learning disability. Methylphenidate (MPH) with behavioural management is associated with benefit in more than half of cases and is well tolerated.
How the study was done:
A structured retrospective case-note review of patients with treatment-resistant epilepsies and IQ < 70 treated for DSM-IV ADHD at a specialist residential epilepsy centre between 1998 and 2005.
Therapeutic interventions:
Treatment with a behavioural management program plus MPH 0.3 - 1 mg/kg/day.
Clinical outcomes:
 Patients (n=18, 13 male) were diagnosed with ADHD at a median age 11.5 years.
 ADHD symptoms improved in 61% (11/18) on the clinical global impressions score.
 Treatment response was not influenced by type of epilepsy.
 Methylphenidate was tolerated by most patients. Adverse effects were mainly
 affective, occurring in 18%.
 There was no statistical evidence for a deterioration of seizure control.
Fosi et al, 2013, DOI: 10.1111/epi.12399
This material is copyrighted by the author(s) and provided as a courtesy for non-commercial educational use
